# Supplementary material for: Free-space optical communications at 4 Gbit/s data rate with a terahertz laser
Source: Commun Phys. 2026 Feb 4;9(1):33. doi: 10.1038/s42005-025-02471-w (PMC12915521; doi:10.1038/s42005-025-02471-w)
Supplement: Supplementary file 2 — Supplementary Information [file 42005_2025_2471_MOESM2_ESM.pdf]

Supplementary Information: Free-space Optical Communications at 4 Gbit/s Data Rate with a Terahertz Laser

Jayaprasath Elumalai[1,*], Mohammed Salih[1], Martyn Fice[2], Adam Brown[1], Lianhe Li[1], Edmund H. Linfield[1], Alexander Valavanis[1], Alwyn J. Seeds[2], Alexander Giles Davies[1] and Joshua R. Freeman[1,*]

[1] School of Electronic and Electrical Engineering, University of Leeds, Leeds, LS2 9JT, UK.

[2] Department of Electronic and Electrical Engineering, University College London, Torrington Place, London, WC1E 7JE, UK.

[*]j.elumalai@leeds.ac.uk; j.r.freeman@leeds.ac.uk

List of Figures

S1 Schematic diagram showing the emission frequencies of the QCL. 2
S2 QCL modes and associated IF peak power at a drive current of 300 mA. . 3
S3 Power spectral density of the receiver system 4
S4 S11 measurement at the QCL 4
S5 BER performance maps as a function of QCL drive current and modulation power evaluated in a THz FSO communication system. 5
S6 NRZ-OOK signal transmission with data rates of 2 Gbit/s, 3 Gbit/s and 4 Gbit/s 7

List of Tables

S1 System-level noise/SNR and power budget analysis 6

Supplementary Note 1: QCL Emission Frequencies

Figure S1 shows the experimental arrangement used to obtain the QCL emission frequencies. The emission frequencies of the QCL were measured by mixing the QCL emission with the 4th harmonic of the amplifier-multiplier-chain (AMC) on the Schottky barrier diode mixer. The RF signal for the LO chain originates from a low phase noise (-76 dBc/Hz @ 100 Hz offset) frequency synthesizer (Keysight N5173B). The RF signal is coupled into a Virginia Diodes $\times$54 AMC (SXG-M WM-380), whose output power at $\sim$600 GHz is controlled by adjusting the AMC input drive power. The Schottky barrier diode harmonic mixer is engineered to operate at the 4th harmonic of the $\sim$600 GHz input signal [1]. A rectangular WM-86 waveguide directs the RF signal to the harmonic mixer,

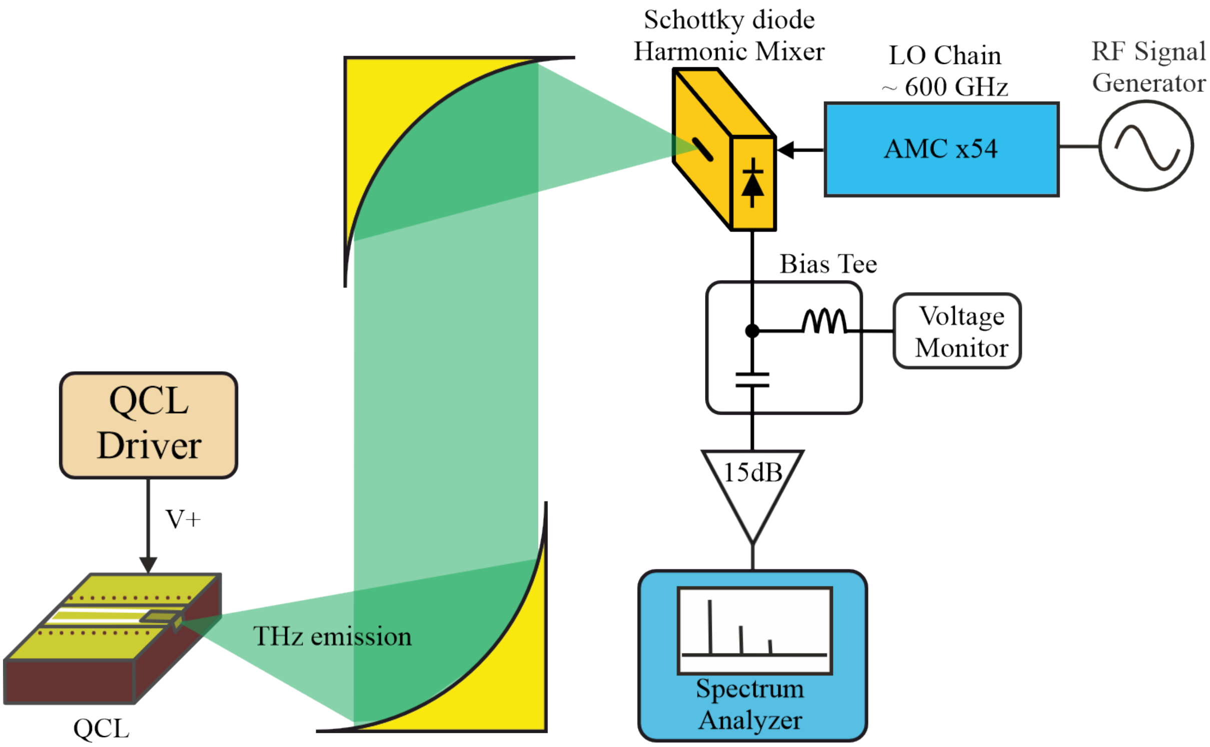

Figure S1: Schematic diagram showing experimental arrangement for the QCL emission frequency measurements. The Schottky barrier diode harmonic mixer generates the fourth harmonic (at 2-3 THz) of the LO frequency, along with the IF signal of the harmonics and the QCL frequency.

while a WM-380 waveguide is integrated into the block for the LO signal. A diagonal horn antenna with a $384 \times 384\,\mu\text{m}^2$ aperture is incorporated into the mixer block to couple the input signal from the QCL into the harmonic mixer [2]. The intermediate frequency was recorded on a low-noise electrical spectrum analyzer (Rohde & Schwarz FSW3030) after amplification by a +15 dB gain low-noise amplifier (ZVA-213UWX-1+). We calculate the QCL frequency (f_{QCL}) via $f_{QCL} = 4Nf_{LO} \pm f_{IF}$, where f_{LO} is the LO driving frequency, N=4 is the AMC multiplication factor, and f_{IF} is the intermediate frequency. Figure S2 shows the QCL modes at a 300 mA drive current obtained by sweeping the LO frequency from the AMC. The primary QCL mode is at 2.4 THz at 300 mA. The QCL modes range

from 2.38 THz to 2.64 THz as we sweep the LO frequency. The blue trace in Figure S2

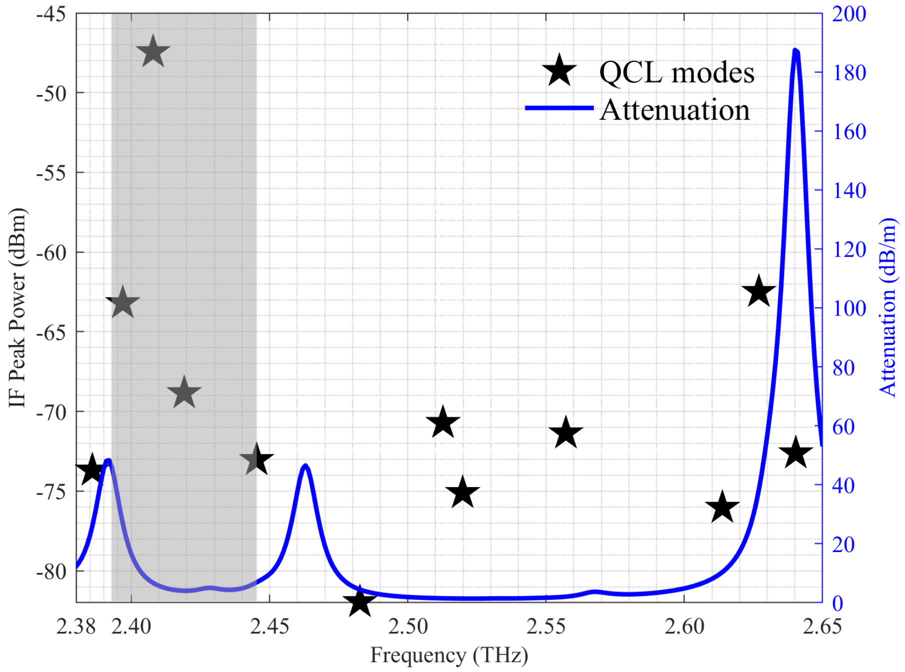

Figure S2: The QCL modes and associated IF peak power at a drive current of 300 mA, highlighting a maximum power difference of +15 dB between the strongest mode near 2.41 THz and the secondary mode around 2.63 THz. The blue trace shows the THz power attenuation over the free-space transmission distance in the frequency range 2.38–2.65 THz. The gray area shows that the primary mode of the QCL, 2.4 THz, is located in a lower attenuation region.

corresponds to the THz power attenuation due to the atmospheric water over the 0.5 m free-space transmission distance in the frequency range between 2.38 THz to 2.65 THz. This was simulated by applying a Voigt line-shape profile to the data cataloged in Ref. [3]. A 40% humidity was assumed in the calculation. A temperature of 293 K was used for Doppler broadening, and a 0.07 $cm^{-1}atm^{-1}$ half-width was assumed for collisional broadening. The frequency window of 2.38–2.64 THz is selected to demonstrate how the QCL modes are influenced by attenuation. The gray area in Figure S2 shows that the primary mode of the QCL at 2.4 THz is located in a lower attenuation region.

Supplementary Note 2: Noise power spectral density and NEP extraction

The noise power spectral density (PSD) of the receiver system (SBD +26.5 GHz bias-tee +15 dB gain RF amplifier) was measured (Figure S3) with THz input blocked, and the same oscilloscope settings were used for data capture. To extract the noise-equivalent power (NEP) of the receiver system (detector and +15 dB gain amplifier), the measured PSD of the noise is first integrated over the measurement bandwidth to obtain the total electrical noise power. Converting this power into an RMS voltage across the 50 Ω load and normalizing by the square root of the measurement bandwidth gives the

output-referred noise spectral density in $V/Hz^{0.5}$. Finally, dividing this noise by the detector responsivity in V/W provides the NEP in $W/Hz^{0.5}$. The total noise power from PSD of 2.99×10^{-9} W, and receiver system responsivity of 176 V/W. The measured total noise over 4 GHz corresponds to a receiver system's NEP of 35 pW/Hz$^{0.5}$. This NEP is

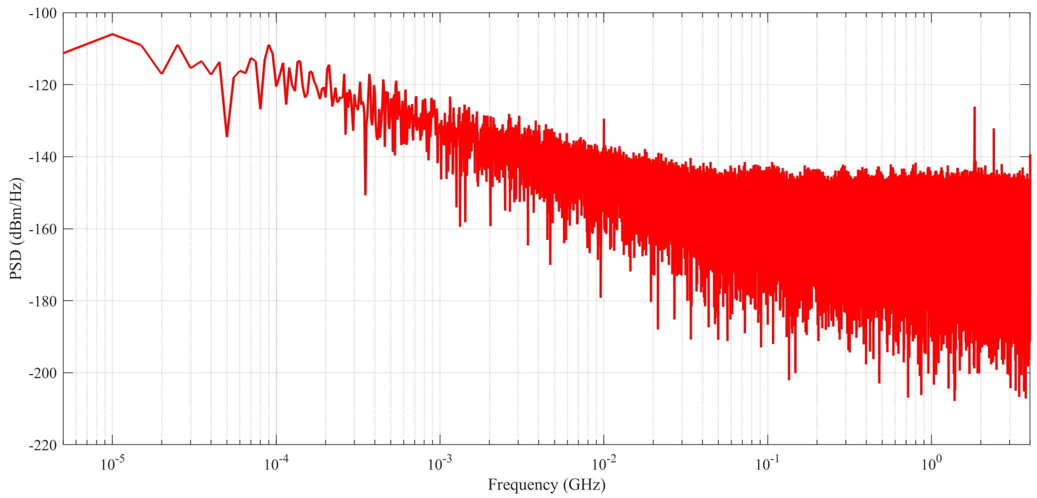

Figure S3: Measured noise of power spectral density of the Schottky diode detector recorded with a +15 dB gain RF amplifier (noise figure is 3 dB) and a 4 GHz oscilloscope.

consistent with other reported values of Schottky diode performance for this frequency range. A zero-bias Schottky detector had NEP ∼17 pW/Hz$^{0.5}$ at 1.2 THz, rising to a few ∼100 pW/Hz$^{0.5}$ at 5.6 THz [4]. Our 2.4 THz NEP lies between those values, as expected.

Supplementary Note 3: Modulation range

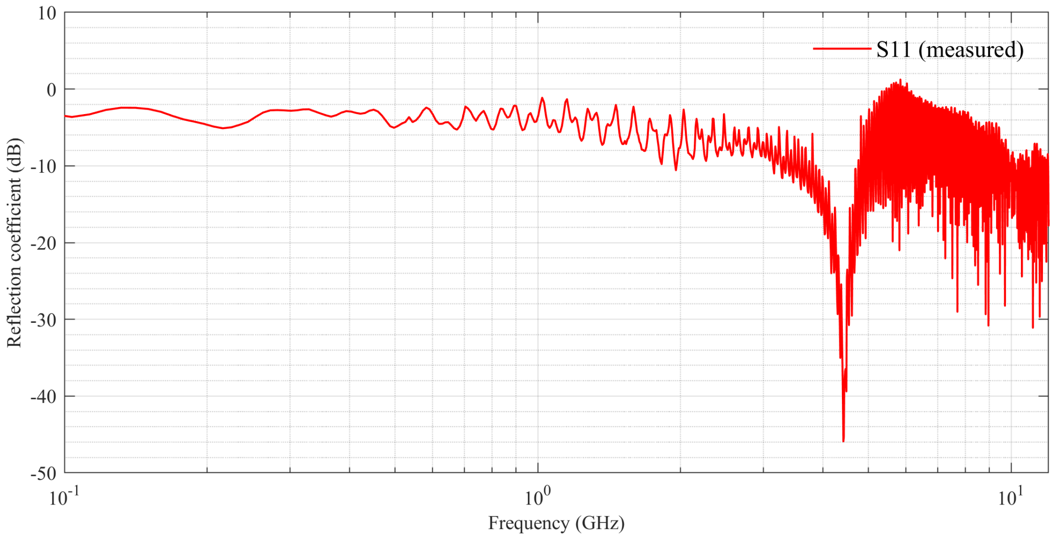

Figure S4: Measured reflection coefficient (S11) at the QCL side of the bias-tee, characterized using a VNA. The measurement covers the frequency range from 0.1 GHz to 12 GHz.

Figure S4 shows the measured reflection coefficient (S11) at the QCL side of a bias-tee, obtained using a VNA. This measurement is essential for characterizing the impedance-matching conditions between the THz QCL transmitter and the RF modulation circuit, which directly influence the quality and efficiency of data modulation in this system.

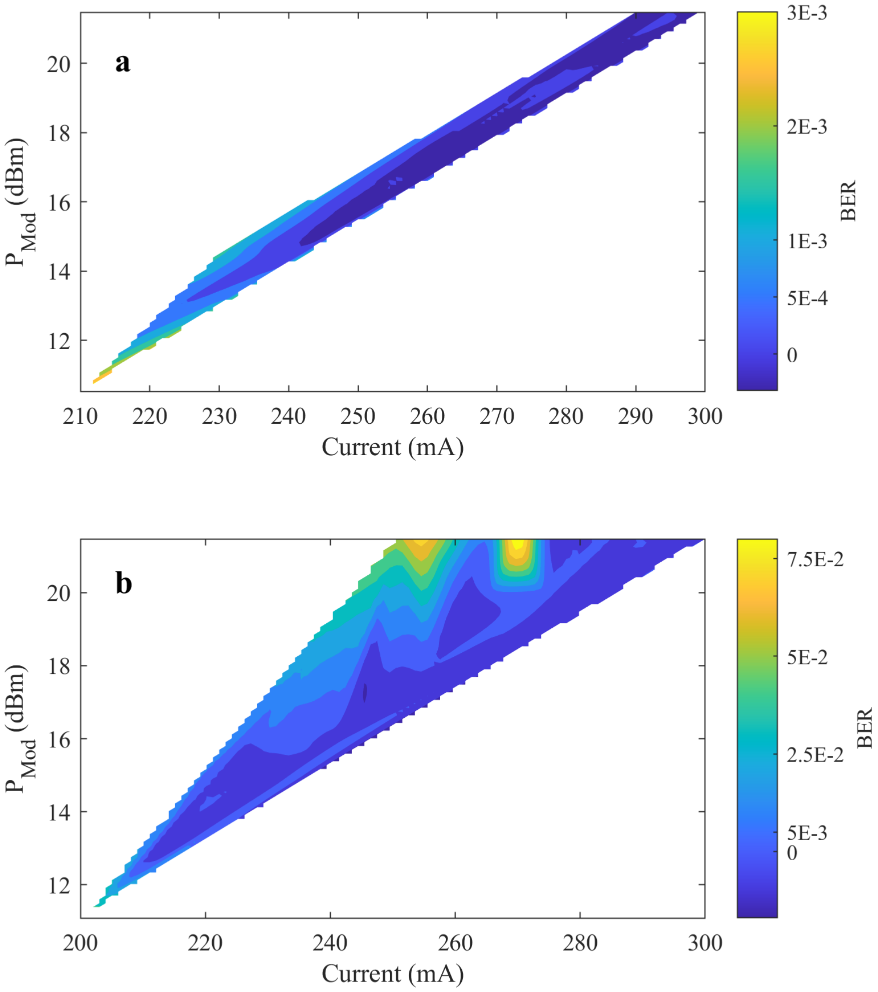

Figure S5: BER performance maps as a function of QCL drive current and modulation power evaluated in a THz FSO communication system. **a** Performance at 1 Gbit/s NRZ-OOK **b** Performance at 2 Gbit/s NRZ-OOK.

In this experiment, the modulation range is fixed by setting the QCL drive current (I) and the modulation power (P_{mod}). The BER of the demodulated signal is evaluated for the NRZ-OOK scheme over a range of drive currents and modulation powers at two different bit rates, 1 Gbit/s (Figure S5a) and 2 Gbit/s (Figure S5b). The BER maps illustrate that in both cases, optimal performance (the lowest BER) is significantly influenced by the combination of $\{I, P_{mod}\}$. The lowest BER (represented by dark blue regions where the BER approaches zero) appears along distinct diagonal bands in both plots. As the drive current changes, the modulation power must be adjusted proportionally to maintain optimal link performance.

In Figure S5a, the best BER performance (below 1×10^{-5}) occurs primarily in a narrow

diagonal region that extends from approximately (220 mA, 12 dBm) to about (300 mA, 19 dBm). The modulation power needs to increase nearly linearly with increasing drive current to maintain a low BER. This indicates that as the drive current is increased, more P_{mod} is required to maintain the optimal modulation depth and SNR. In Figure S5b, the optimal region (shown in dark blue, representing the lowest BER) becomes narrower and less uniform, highlighting the stricter operational conditions needed at this larger bit rate. Regions of significantly increased BER ($>7.5 \times 10^{-2}$) appear prominently when P_{mod} and drive current are misaligned, especially around (260 mA, ∼19 dBm). This suggests that higher modulation speeds necessitate stricter P_{mod} adjustments for specific drive currents to maintain system performance. In our experiment, we fixed the QCL drive current at 300 mA and, based on the analysis above, set the modulation power to 19 dBm to achieve good system transmission performance. The modulation power, equivalent to 48 mA rms current in QCL operation, is calculated using the following equation $I_{\mathrm{mA}} = \sqrt{(10^{P_{\mathrm{dBm}}/10})/(1000 \times Z_{\mathrm{QCL}})}$ where Z_{QCL} is the measured impedance of the QCL is 35 Ω.

Supplementary Note 4: Link power budget

The power link budget and SNR for the 2.4 THz FSO link are calculated using the measured receiver system parameters and known transmitter characteristics. At the receiver focus, we measured P_{THz} approximately 75 µW at a drive current of 300 mA and a temperature of 19 K. With a modulation of 19 dBm applied, we estimate a modulated power of 50 µW. The expected output amplitudes and SNR at this modulated power are

Table S1: System-level noise/SNR and power budget analysis

**Modulated Power** (P_{mod})	50 µW
**Bandwidth** (B)	4 GHz
**Receiver system Responsivity**	176 V/W
**System NEP**	35 pW/$\sqrt{\mathrm{Hz}}$
**SNR** ($P_{\mathrm{mod}}/(\mathrm{NEP}\times\sqrt{B})$)	13.6 dB

listed in Table S1. The system NEP of 35 pW/$\sqrt{\mathrm{Hz}}$ is obtained from the measured noise PSD and with the receiver system responsivity of ∼176 V/W. The end-to-end system's 3-dB bandwidth of ∼5 GHz (measured) sets the modulation bandwidth limitation, while the NEP sets the power margin. Overall, the link budget analysis indicates that the system operates with a satisfactory SNR at 4 Gbit/s over 0.5 m. Future improvements (higher QCL power or a more sensitive receiver) could allow longer ranges or higher data rates.

Supplementary Note 5: NRZ-OOK signal transmission with data rates of 2 Gbit/s, 3 Gbit/s, and 4 Gbit/s

Figure S6 illustrates the performance of the THz FSO communication system for the NRZ-OOK message signal at data rates of 2 Gbit/s [Figure S6(i)], 3 Gbit/s [Figure S6(ii)],

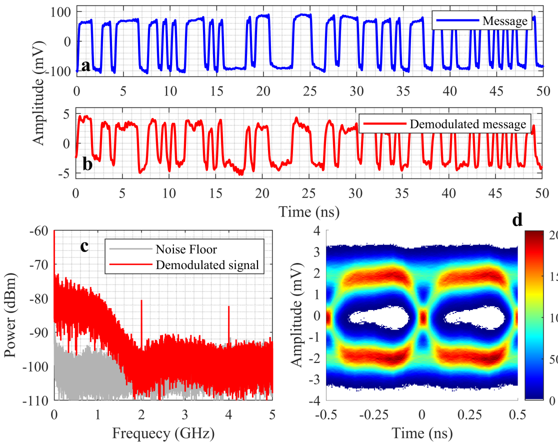

(i) 2 Gbit/s

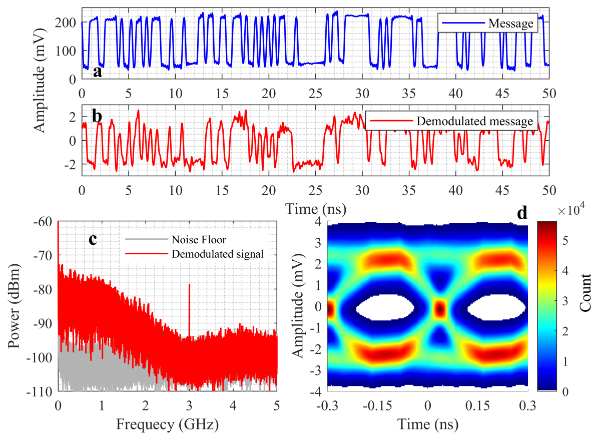

(ii) 3 Gbit/s

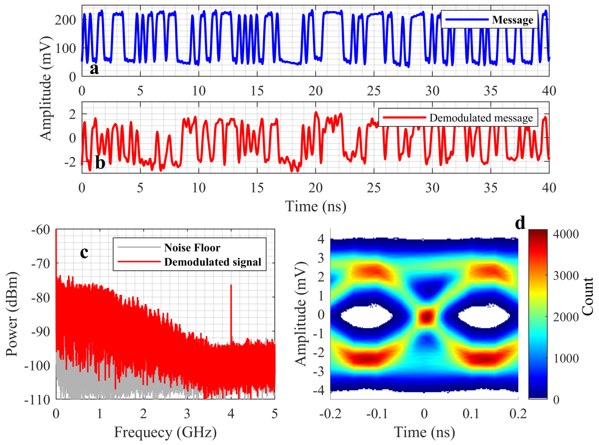

(iii) 4 Gbit/s

Figure S6: NRZ-OOK signal transmission with data rates of (i) 2 Gbit/s, (ii) 3 Gbit/s and (iii) 4 Gbit/s. **a** The transmitted NRZ-OOK message signal showing clean binary transitions generated by the AWG. **b** The demodulated signal received after 0.5 m of free-space transmission, demonstrating successful recovery of the transmitted waveform with minimal distortion. **c** Power spectrum of the demodulated signal (red) and the noise floor (gray trace) when the QCL is turned off. **d** The eye diagram is plotted across the bit interval and received signal amplitude, indicating the levels of the "0" and "1" bits. The color bar represents the hit count of the signal at each point in the eye. For all data rates, the eye diagrams exhibit a clear pattern, validating the reliability of the THz FSO communication system for high-speed transmission of NRZ-OOK signals.

and 4 Gbit/s [Figure S6(iii)]. The QCL is biased at 300 mA, and has an output power of 75 μW. The modulated signal is transmitted over 0.5 m in free-space before reaching the receiver. As shown in Figure S6, the demodulated signals maintain sufficient accuracy with minimal distortion to ensure reliable data transmission for all three data rates.

The power spectra of the demodulated signals provide further insights into the NRZ-OOK transmission in the frequency domain. For example, in Figure S6(i), the minima in the spectra at 2 and 4 GHz are the most significant features indicating NRZ-OOK modulation for 2 Gbit/s. The sharp peaks at 2 and 4 GHz reflect distortion in the received signal. The NRZ-OOK signal is a form of rectangular pulse modulation, which inherently

produces higher-order harmonics due to the sharp transitions between the “0” and “1” bits. The broad peaks between the minima decrease in optical power as a function of frequency, reflecting the pulse shape. Due to the finite rise and fall times, their amplitudes reduce more rapidly compared to perfectly square pulses.

The eye diagrams in Figure S6 were plotted using a total of 10^7 symbols over a 2 ms time span of the received signal without filtering the signal. It can provide critical insights into system performance, including timing jitter, inter-symbol interference, and signal quality. The eye diagrams are plotted across the bit interval, capturing the temporal characteristics of the transmitted data stream and received signal amplitude, indicating the levels of the “0” and “1” bits. The color scale represents the density of the signal traces, with warmer colors (red/yellow) corresponding to higher counts and cooler colors (blue) denoting lower count regions. The diagrams exhibit clear eyes with well-defined transitions between “0” and “1” bits, indicating minimal distortion and inter-symbol interference. The clarity of the eye pattern highlights the system’s ability to maintain timing precision and amplitude integrity over the evaluated free-space transmission path.

References

[1] Reck, T. J., Durant, S. & Hesler, J. L. Design of a 2.5 THz Schottky-diode fourth-harmonic mixer. *IEEE Trans. THz Sci. Technol.* **13**, 580–586 (2023).

[2] Johansson, J. F. & Whyborn, N. D. The diagonal horn as a sub-millimeter wave antenna. *IEEE Trans. Microw. Theory Tech.* **40**, 795–800 (1992).

[3] Pickett, H. M. *et al.* Submillimeter, millimeter, and microwave spectral line catalog. *J. Quant. Spectrosc. Radiat. Transf.* **60**, 883–890 (1998).

[4] Yadav, R. *et al.* State-of-the-art room temperature operable zero-bias Schottky diode-based terahertz detector up to 5.56 THz. *Sensors* **23**, 3469 (2023).
